# Differences in fungal communities in the fur of two- and three-toed sloths revealed by ITS metabarcoding

**DOI:** 10.1099/mic.0.001309

**Published:** 2023-02-27

**Authors:** Diego Rojas-Gätjens, Judy Avey-Arroyo, Priscila Chaverri, Keilor Rojas-Jimenez, Max Chavarría

**Affiliations:** ^1^​ Centro Nacional de Innovaciones Biotecnológicas (CENIBiot), CeNAT-CONARE, 1174-1200, San José, Costa Rica; ^2^​ The Sloth Sanctuary of Costa Rica, Limon, Costa Rica; ^3^​ Escuela de Biología, Universidad de Costa Rica, 11501-2060, San José, Costa Rica; ^4^​ Centro de Investigaciones en Productos Naturales (CIPRONA), Universidad de Costa Rica, 11501-2060, San José, Costa Rica; ^5^​ Escuela de Química, Universidad de Costa Rica, 11501-2060, San José, Costa Rica

**Keywords:** Ascomycota, Basidiomycota, Capnodiales, *Cladosporium*, *Neodevriesia*, sloths

## Abstract

Sloths have dense fur on which insects, algae, bacteria and fungi coexist. Previous studies using cultivation-dependent methods and 18S rRNA sequencing revealed that the fungal communities in their furs comprise members of the phyla Ascomycota and Basidiomycota. In this note, we increase the resolution and knowledge of the mycobiome inhabiting the fur of the two- (*Choloepus hoffmanni*) and three-toed (*Bradypus variegatus*) sloths. Targeted amplicon metagenomic analysis of ITS2 nrDNA sequences obtained from 10 individuals of each species inhabiting the same site revealed significant differences in the structure of their fungal communities and also in the alpha-diversity estimators. The results suggest a specialization by host species and that the host effect is stronger than that of sex, age and animal weight. Capnodiales were the dominant order in sloths’ fur and *Cladosporium* and *Neodevriesia* were the most abundant genera in *Bradypus* and *Choloepus*, respectively. The fungal communities suggest that the green algae that inhabit the fur of sloths possibly live lichenized with Ascomycota fungal species. The data shown in this note offer a more detailed view of the fungal content in the fur of these extraordinary animals and could help explain other mutualistic relationships in this complex ecosystem.

## Full-Text

Previous studies have shown that sloths’ fur is a complex ecosystem where insects, algae, fungi and bacteria coexist [1–4]. Some of these organisms have been reported to live in symbiotic relationships with the sloth (e.g. the green algae *Trichophilus*) [4]. However, most of the biological interactions of this ecosystem remain unknown. The study of micro-organisms in the fur of sloths has been of particular interest not only to understand the ecology of these animals, but also because they are a source of bioactive molecules.

Recently, our group elucidated the presence of antibiotic-producing bacteria in the fur of two- (*Choloepus hoffmanni*) and three-toed (*Bradypus variegatus*) sloths [2]. Higginbotham *et al*. [3] isolated 84 fungi from the fur of *B. variegatus,* which all belonged to the phylum Ascomycota, and the 2 most common genera were *Pestalotiopsis* and *Trichoderma*. Furthermore, these authors demonstrated that many of them secrete bioactive compounds. In addition, Suutari *et al*. [4] studied the diversity of the eukaryotic community present in the fur of six sloth species from Central and South America via the generation of clone libraries of the 18S rRNA. The authors focused on the study of green algal communities; however, their results also revealed that the fungal communities were governed by members of the Ascomycota and Basidiomycota.

**Fig. 1.**
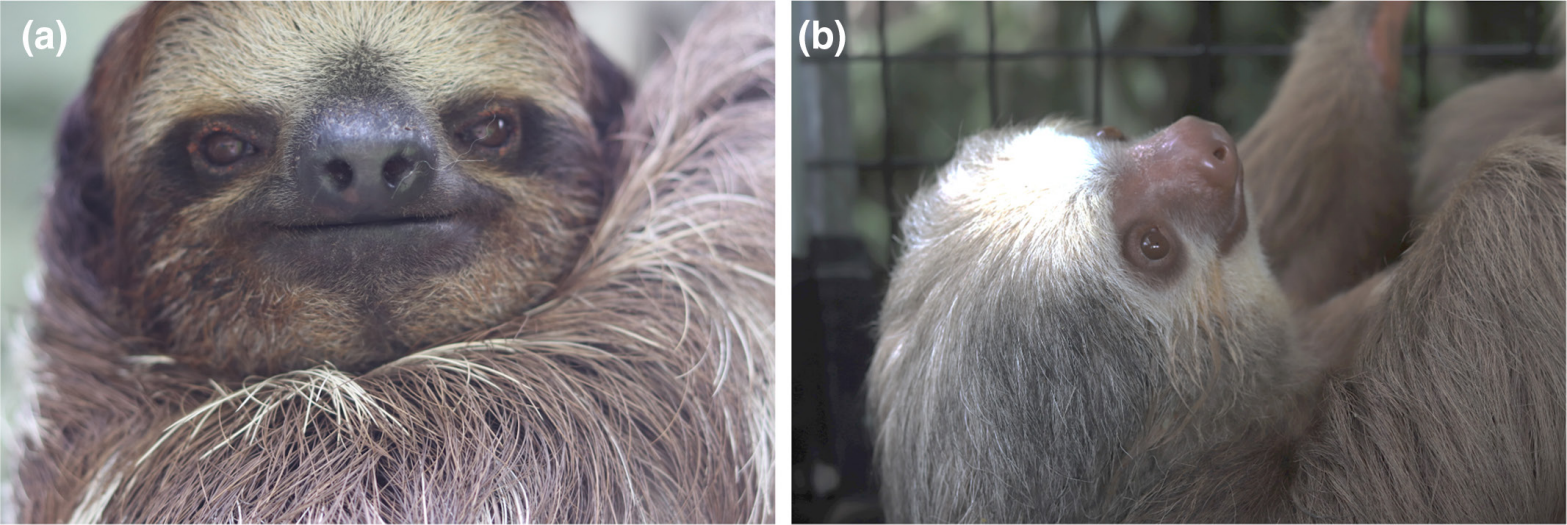
Sloths that inhabit in the Sloth Sanctuary of Costa Rica located in Cahuita, Costa Rica. (**a**) *Bradypus variegatus* and (**b**) *Choloepus hoffmanni*.

Here, we have proposed to expand the picture of the mycobiota that inhabits the fur of sloths by sequencing the ITS2 region of the nuclear ribosomal DNA through metabarcoding in the species *B. variegatus* and *C. hoffmanni* (Fig. 1). This approach will allow us to have a higher resolution of the fungal communities that inhabit these animals.

A total of 20 samples of sloth hair were obtained from the Sloth Sanctuary (http://www.slothsanctuary.com) in Cahuita, Limon, as previously described [2]. Details of the characteristics of the sanctuary and the way in which the animals live there were previously described [2]. DNA extraction of sloth fur was also performed as described by Rojas-Gätjens *et al*. [2]. Subsequently, amplicon libraries were created using the primer pair ITS3-2024F (GCATCGATGAAGAACGCAGC) and ITS4-2409R (TCCTCCGCTTATTGATATGC) [5]. Illumina-sequenced paired-end fastq files were deposited in the National Center for Biotechnology Information (NCBI) Sequence Read Archive (SRA) under PRJNA876237 and processed with DADA2 version 1.2.0. Details of the data processing and statistical analyses are provided in the (File S1, available in the online version of this article).

**Fig. 2.**
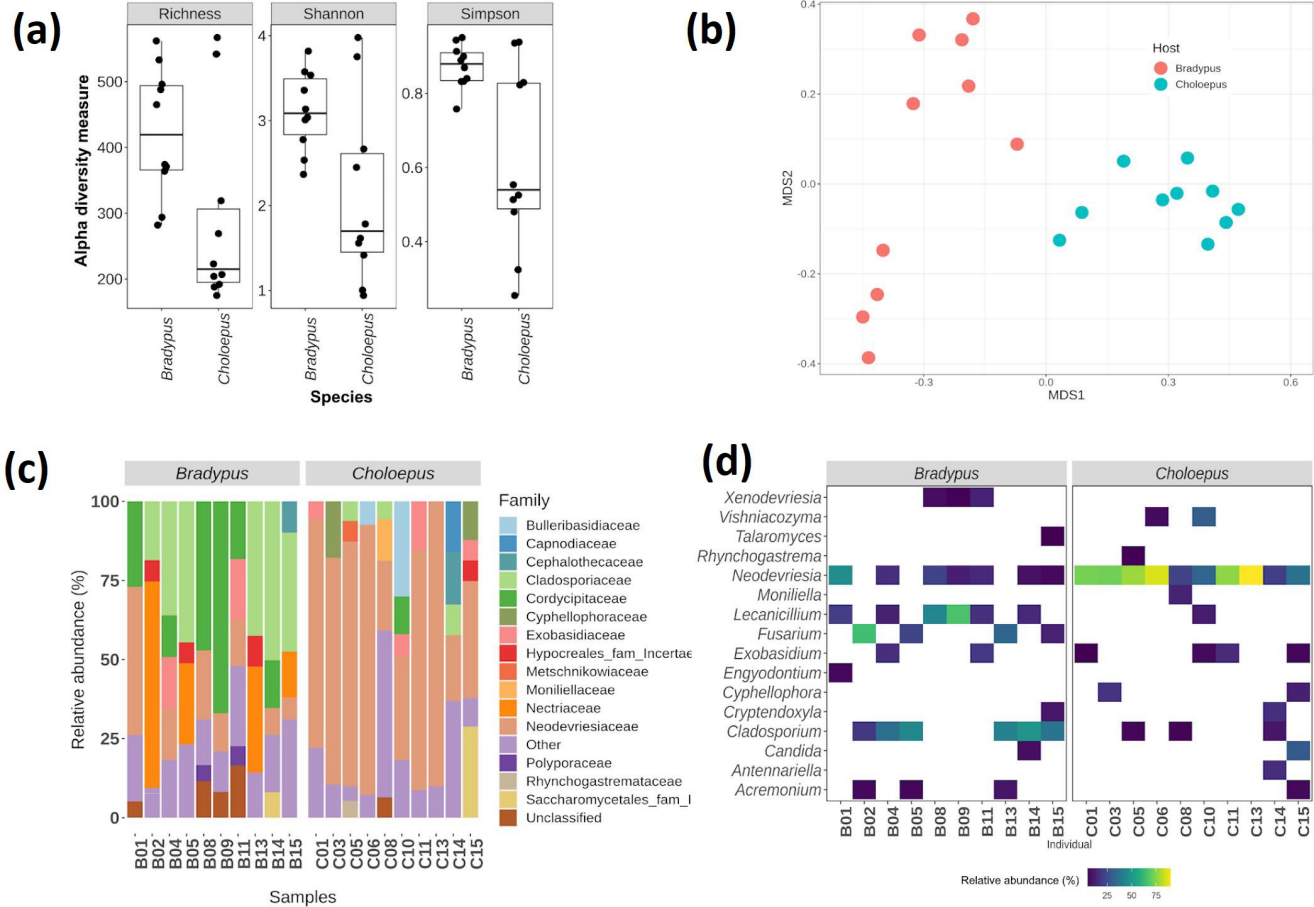
Mycobiome of the fur of three- and two-toed sloths. (**a**) Diversity measures of the hair samples from *B. variegatus* and *C. hoffmanni*. The diversity measures (Shannon, Simpson and observed richness) were calculated using phyloseq. (**b**) NMDS analysis of the fungal communities in the hair of both sloth species. (**c**) Taxonomic composition at the family level of fungal community inhabiting the hair of *B. variegatus* and *C. hoffmanni*. (**d**) Taxonomic composition of fungal community inhabiting the hair of *B. variegatus* and *C. hoffmanni* at the genus level across the 20 samples analysed (*x*-axis).

We obtained a total of 3164 fungal amplicon sequence variants (ASVs) (Table S1). Our results showed significant statistical differences (richness, *P*=0.02881; Shannon, *P*=0.03546; Simpson, *P*=0.01469) in the diversity of the fungal communities inhabiting the two sloth species. All diversity estimators indicate a higher fungal diversity in *B. variegatus* than in *C. hoffmanni* (see Fig. 2a). All ASVs detected in both sloth species belong to the phyla Ascomycota and Basidiomycota (Table S1). These results are consistent with what was reported by Higginbotham *et al*. [3], where all isolates obtained in *Bradypus* sloths belonged to the Ascomycota. It is also consistent with Suutari *et al*. [4], which identified the presence of Ascomycota and Basidiomycota in sloths using the 18S rRNA marker.

The non-metric multidimensional scaling (NMDS) and PERMANOVA analyses revealed differences in the structure of the fungal communities inhabiting the fur of both species of sloths (PERMANOVA, *P*=0.001) (Fig. 2b). These results suggest a possible level of specialization of the host’s mycobiota. Within the species *B. variegatus,* two differentiated clusters were observed. We performed additional statistical analyses to determine the weight of other variables in the observed differences. However, it seems that the host effect is the only variable that shows a significant difference (tested variables: sex, years in the sanctuary and animal weight when received) (Fig. S1).

Capnodiales was the most abundant order in both sloth species (*Bradypus* 20.6–60.3 %; *Choloepus* 33.7–91.5 %). Capnodiales is the second largest order in the class Dothideomycetes [6] and its members have been reported as plant pathogens [7, 8], on lichens [6], in hot springs [9], in marine environments [10] and on mammal skin [11, 12]. These fungi have been reported as being most abundant in the dog and rat skin mycobiota [11, 12]. Their presence has also been reported in other animals, such as snakes [13], salamanders [14] and parrots [15], often associated with skin lesions.

At the family level (Fig. 2c), *Choloepus* sloths were dominated by fungi of the *Neodevriesiaceae* (20.8–90.1 %). This family was also present in *Bradypus* but with lower abundance (7.1–46.8 %). Most of the *Neodevriesiaceae* ASVs found in the samples belonged to *Neodevriesia* (Fig. 2d)*,* a genus segregated from *Cladosporium* [16]. This genus is commonly found in marine environments [16, 17], including causing fish lesions [18].

In contrast, *Bradypus* is dominated by members of the *Cladosporiaceae* (18.6–50.2 %) and *Cordycipitaceae* (13.2–66.9 %). These families were not very abundant in *Choloepus* samples. Cladosporiaceae was mainly represented by the genus *Cladosporium* (Fig. 2d). *Cladosporium* has been reported to be a highly diverse genus found in most non-extreme habitats around the globe, including plants, animals and soils [19, 20]; nevertheless, they are particularly abundant in indoor environments. Some species are important in human health, particularly in allergic lung mycoses [21]. On the other hand, most members of the *Cordypitaceae* were classified in *Lecanicillium*. This genus is a known entomopathogen that infects a great diversity of arthropods, including aphids, whiteflies and Lepidoptera larvae [22, 23]. The presence of this micro-organism is probably associated with the high prevalence of arthropods in the sloths’ fur and could be involved in the decomposition of moths proposed by Pauli *et al*. [1]. These authors suggested that Ascomycota are responsible for mineralizing the moths to increase the inorganic nitrogen levels, which helps the algae grow. Later, these algae are consumed by sloths to enrich their limited diet and secondarily to collaborate with the animal’s camouflage.

In addition to what was previously proposed, we consider that there could also be a symbiotic relationship between the algae and some species in Ascomycota. Previous research has shown that taxa in the Ascomycota (e.g. members of the orders Helotiales, Capnodiales, Peltigerales and Verrucariales, among others) are commonly found in symbiotic relationships with algae forming lichens [24–28]. For example, *Cladosporium* associates with red algae (*Porphyra yezoensis*) [29] and marine brown algae (*Actinotrichia fragilis*) [30] to form lichens. *Neodevriesia,* one of the most abundant genera found in *Choloepus*, has also been reported to form lichens with marine algae [16].

The classic way this symbiotic relationship has been explained proposes that the fungus contributes to the protection of the algae against desiccation and radiation, while the algae produce nutrients photosynthetically for the fungus [31]. Therefore, it is reasonable to think that the fungal communities could have a more complex relationship with the green algae in addition to the previously assigned role as moth decomposer in sloth fur [1]. Our results suggest that the green algae that inhabit the fur of sloths possibly live lichenized with Ascomycota fungal species, an idea that has also been suggested by Kaup *et al*. [32].

Several questions arise from the results of our study, and one of them is whether the differences observed in the fungal community’s structure could explain the differences in the algal growth observed between sloth species (*Bradypus* tends to form more algal biomass) [1]. The data shown in this note help to shed light on the mycobiota inhabiting the fur of three- and two-toed sloths, revealing differences in their structure and offering a more detailed view of the fungal content in the fur of these extraordinary animals.

## Supplementary Data

Supplementary material 1

Supplementary material 2

Supplementary material 3

## References

[1] Pauli JN, Mendoza JE, Steffan SA, Carey CC, Weimer PJ (2014). A syndrome of mutualism reinforces the lifestyle of a sloth. Proc Biol Sci.

[2] Rojas-Gätjens D, Valverde-Madrigal KS, Rojas-Jimenez K, Pereira R, Avey-Arroyo J (2022). Antibiotic-producing Micrococcales govern the microbiome that inhabits the fur of two- and three-toed sloths. Environ Microbiol.

[3] Higginbotham S, Wong WR, Linington RG, Spadafora C, Iturrado L (2014). Sloth hair as a novel source of fungi with potent anti-parasitic, anti-cancer and anti-bacterial bioactivity. PLoS One.

[4] Suutari M, Majaneva M, Fewer DP, Voirin B, Aiello A (2010). Molecular evidence for a diverse green algal community growing in the hair of sloths and a specific association with *Trichophilus welckeri* (Chlorophyta, Ulvophyceae). BMC Evol Biol.

[5] Toju H, Tanabe AS, Yamamoto S, Sato H (2012). High-coverage ITS primers for the DNA-based identification of Ascomycetes and Basidiomycetes in environmental samples. PLoS One.

[6] Abdollahzadeh J, Groenewald JZ, Coetzee MPA, Wingfield MJ, Crous PW (2020). Evolution of lifestyles in *Capnodiales*. Stud Mycol.

[7] Abdelfattah A, Cacciola SO, Mosca S, Zappia R, Schena L (2017). Analysis of the fungal diversity in citrus leaves with greasy spot disease symptoms. Microb Ecol.

[8] Batzer JC, Sisson AJ, Harrington TC, Mayfield DA, Gleason ML (2012). Temporal patterns in appearance of sooty blotch and flyspeck fungi on apples. Microb Ecol.

[9] Yamazaki A, Toyama K, Nakagiri A (2010). A new acidophilic fungus *Teratosphaeria acidotherma* (Capnodiales, Ascomycota) from a hot spring. Mycoscience.

[10] Gnavi G, Ercole E, Panno L, Vizzini A, Varese GC (2014). Dothideomycetes and Leotiomycetes sterile mycelia isolated from the Italian seagrass *Posidonia oceanica* based on rDNA data. Springerplus.

[11] Tang S, Prem A, Tjokrosurjo J, Sary M, Van Bel MA (2020). The canine skin and ear microbiome: a comprehensive survey of pathogens implicated in canine skin and ear infections using a novel next-generation-sequencing-based assay. Vet Microbiol.

[12] Sanjar F, Weaver AJ, Peacock TJ, Nguyen JQ, Brandenburg KS (2020). Temporal shifts in the mycobiome structure and network architecture associated with a rat (*Rattus norvegicus*) deep partial-thickness cutaneous burn. Med Mycol.

[13] Dubey S, Pellaud S, Gindro K, Schuerch J, Golay J (2022). Fungal infection in free-ranging snakes caused by opportunistic species. EAS.

[14] García-Sánchez JC, Arredondo-Centeno J, Segovia-Ramírez MG, Tenorio Olvera AM, Parra-Olea G (2022). Factors influencing bacterial and fungal skin communities of montane salamanders of Central Mexico. Microb Ecol.

[15] Krumbeck JA, Turner DD, Diesel A, Hoffmann AR, Heatley JJ (2022). Skin microbiota of quaker parrots (*Myiopsitta monachus*) with normal feathering or feather loss via next-generation sequencing technology. J Exot Pet Med.

[16] Wang M-M, Shenoy BD, Li W, Cai L (2017). Molecular phylogeny of *Neodevriesia*, with two new species and several new combinations. Mycologia.

[17] Lee LC, Rizman-Idid M, Alias SA, Palaniveloo K, Gu H (2022). First record of the fungal genus *Neodevriesia* Quaedvl. & Crous (Ascomycota, Dothideomycetes, Neodevriesiaceae) isolated from scleractinian corals of Perhentian Islands, Malaysia. Biodivers Data J.

[18] Armwood AR, Cañete-Gibas CF, Dill-Okubo JA, Wiederhold NP, Camus AC (2021). Retrospective study of phaeohyphomycosis in aquarium-housed fish, with first descriptions of *Exophiala lecanii-corni* and *Neodevriesia cladophorae* in fish. J Fish Dis.

[19] Godinho VM, de Paula MTR, Silva DAS, Paresque K, Martins AP (2019). Diversity and distribution of hidden cultivable fungi associated with marine animals of Antarctica. Fungal Biol.

[20] Zaccaron AZ, Chen LH, Samaras A, Stergiopoulos I (2022). A chromosome-scale genome assembly of the tomato pathogen *Cladosporium fulvum* reveals a compartmentalized genome architecture and the presence of a dispensable chromosome. Microb Genom.

[21] Bensch K, Braun U, Groenewald JZ, Crous PW (2012). The genus *Cladosporium*. Stud Mycol.

[22] Su L, Zhu H, Guo Y, Du X, Guo J (2019). *Lecanicillium coprophilum* (Cordycipitaceae, Hypocreales), a new species of fungus from the feces of *Marmota monax* in China. Phytotaxa.

[23] Vu VH, Hong SI, Kim K (2007). Selection of entomopathogenic fungi for aphid control. J Biosci Bioeng.

[24] Spribille T, Tuovinen V, Resl P, Vanderpool D, Wolinski H (2016). Basidiomycete yeasts in the cortex of ascomycete macrolichens. Science.

[25] Simon A, Goffinet B, Wang L, Spribille T, Goward T (2022). Global phylogeny and taxonomic reassessment of the lichen genus *Dendriscosticta* (Ascomycota: Peltigerales). Taxon.

[26] Zhang T, Wei XL, Zhang YQ, Liu HY, Yu LY (2015). Diversity and distribution of lichen-associated fungi in the Ny-Ålesund Region (Svalbard, High Arctic) as revealed by 454 pyrosequencing. Sci Rep.

[27] Vicente TFL, Gonçalves MFM, Brandão C, Fidalgo C, Alves A (2021). Diversity of fungi associated with macroalgae from an estuarine environment and description of *Cladosporium rubrum* sp. nov. and *Hypoxylon aveirense* sp. nov. Int J Syst Evol Microbiol.

[28] Pino-Bodas R, Stenroos S (2021). Global biodiversity patterns of the photobionts associated with the genus *Cladonia* (Lecanorales, Ascomycota). Microb Ecol.

[29] Ding L, Qin S, Li F, Chi X, Laatsch H (2008). Isolation, antimicrobial activity, and metabolites of fungus *Cladosporium* sp. associated with red alga *Porphyra yezoensis*. Curr Microbiol.

[30] Shigemori H, Kasai Y, Komatsu K, Tsuda M, Mikami Y (2004). Sporiolides A and B, new cytotoxic twelve-membered macrolides from a marine-derived fungus *Cladosporium* species. Marine Drugs.

[31] Grimm M, Grube M, Schiefelbein U, Zühlke D, Bernhardt J (2021). The lichens’ microbiota, still a mystery?. Front Microbiol.

[32] Kaup M, Trull S, Hom EFY (2021). On the move: sloths and their epibionts as model mobile ecosystems. Biol Rev Camb Philos Soc.

