# Supplementary figures and images for: Differences in fungal communities in the fur of two- and three-toed sloths revealed by ITS metabarcoding
Source: Microbiology (Reading). 2023 Feb 27;169(2):001309. doi: 10.1099/mic.0.001309 (PMC10197869; doi:10.1099/mic.0.001309)

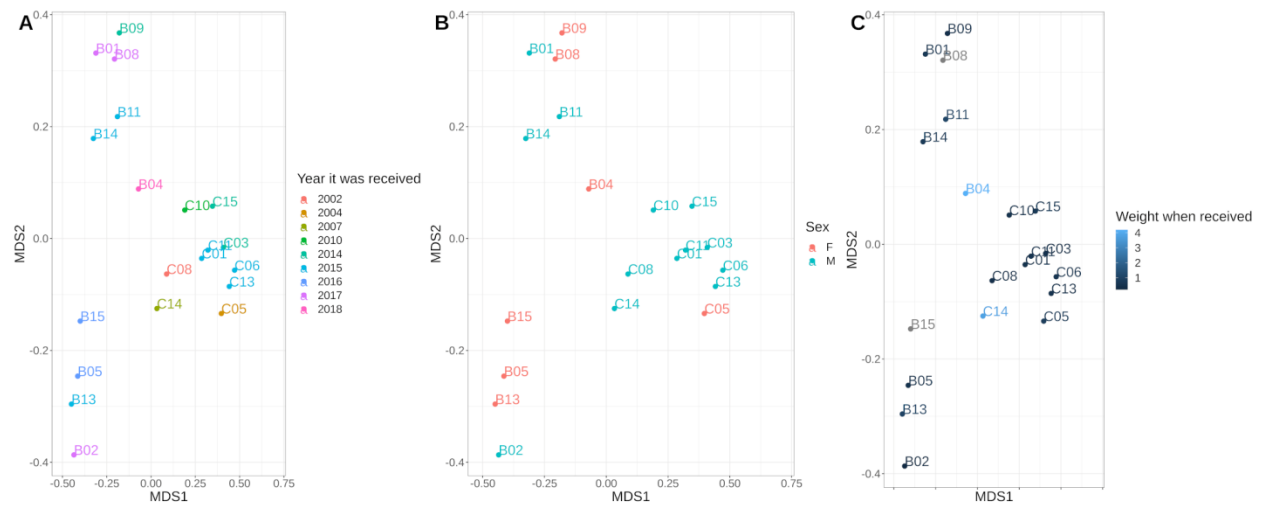

**Fig. S1.** NMDS analysis of the fungal communities in the fur of both sloth species.

Supplement: Supplementary material 1 [file mic-169-1309-s002.pdf]
