# Supplementary material for: Differences in fungal communities in the fur of two- and three-toed sloths revealed by ITS metabarcoding
Source: Microbiology (Reading). 2023 Feb 27;169(2):001309. doi: 10.1099/mic.0.001309 (PMC10197869; doi:10.1099/mic.0.001309)
Supplement: Supplementary material 3 [file mic-169-1309-s004.pdf]

File S1

Differences in fungal communities in the fur of two- and three-toed sloths revealed by ITS metabarcoding

Diego Rojas-Gätjens[1], Judy Avey-Arroyo[2], Priscila Chaverri[3,4], Keilor Rojas-Jimenez[3*] & Max Chavarría[1,4,5*]

[1]Centro Nacional de Innovaciones Biotecnológicas (CENIBiot), CeNAT-CONARE, 1174-1200, San José, Costa Rica. [2]The Sloth Sanctuary of Costa Rica, Limon, Costa Rica. [3]Escuela de Biología, Universidad de Costa Rica, 11501-2060, San José, Costa Rica. [4]Centro de Investigaciones en Productos Naturales (CIPRONA), Universidad de Costa Rica, 11501-2060, San José, Costa Rica. [5]Escuela de Química, Universidad de Costa Rica, 11501-2060, San José, Costa Rica.

Running title: Fungi in the fur of two- and three-toed sloths.

Keywords: Ascomycota, Basidiomycota, Capnodiales, *Cladosporium, Neodevriesia,* sloths.

---

* Correspondence to:

Max Chavarría

Escuela de Química & Centro de Investigaciones en Productos Naturales (CIPRONA)

Universidad de Costa Rica, Sede Central, San Pedro de Montes de Oca, San José, 11501-2060, Costa Rica

Phone (+506) 2511 8520. E-mail: max.chavarria@ucr.ac.cr

ORCID: https://orcid.org/0000-0001-5901-3576

Keilor Rojas-Jiménez

Escuela de Biología, Universidad de Costa Rica

Sede Central, San Pedro de Montes de Oca, San José, 11501-2060, Costa Rica

Phone (+506) 2511 5871

ORCID: https://orcid.org/0000-0003-4261-0010

SUPPLEMENTARY METHODS

We used the DADA2 version 1.21.0 to process the Illumina-sequenced paired-end fastq files and generate a table of amplicon sequence variants (ASVs) [32]. Briefly, we removed primers and adapters, inspected the quality profiles of the reads, filtered and trimmed sequences with a quality score < 30, estimated error rates, modeled and corrected amplicon errors, and inferred the sequence variants. Then, we merged the forward and reverse reads to obtain the full denoised sequences, removed chimeras, and constructed the ASV table. We assigned taxonomy to the ASVs with the function assignTaxonomy, of DADA2, which uses as input the set of sequences to be classified and a training set of reference sequences with known taxonomy, which in this case was the UNITE ITS database version 8.3 [33]. We carried out a second taxonomic assignment of the ASVs using the tool IDTAXA in DECIPHER [34] with the same version of UNITE as a reference and a confidence threshold >60%, and additionally, a third classification using the Classifier tool [35] implemented in the Ribosomal Database Project (http://rdp.cme.msu.edu/) using as reference the Warcup Fungal ITS trainset V2 database [36]. The consistency between the different programs' taxonomic assignments was verified, followed by performing a manual curation. In cases of discrepancies, comparisons with the BLAST tool of NCBI Genbank was also used. All ASVs that appeared only once (singletons) in the dataset were discarded. Sequence data were deposited into the NCBI Sequence Read Archive under PRJNA876237.

The Statistical analyses and the visualization of results were performed with the R statistical program (R-Core-Team 2022) and the Rstudio interface. Package Vegan v2.5.7 [37] was used to calculate alpha diversity estimators (Shannon, Simpson and Richness). Comparisons of the alpha diversity estimators between sloth's species were performed using a Wilcoxon test. For non-metric multidimensional scaling analyses (NMDS) data tables with the amplicon sequence variants (ASV) abundances were normalized into relative abundances and converted into a Bray–Curtis similarity matrix, then the NMDS analysis was performed. The similarity matrix of both sloths species were compared using PERMANOVA (function adonis2). Furthermore, differences in the communities of the same sloth specie was evaluated according to sex, year it was received in the sanctuary and weight when it was received using visual analysis (NMDS), nevertheless no clear clustering was observed.

REFERENCES

[32] Callahan B, McMurdie P, Holmes S (2017) Exact sequence variants should replace operational taxonomic units in marker-gene data analysis. *ISME J* 11:2639–2643. https://doi.org/10.1038/ismej.2017.119

[33] Abarenkov K, Zirk A, Piirmann T, Pöhönen R, Ivanov F, Nilsson RH, Urmas K (2021) UNITE general FASTA release for Fungi. UNITE Community. 10.15156/BIO/1280049

[34] Murali A, Bhargava A, Wright ES (2018) IDTAXA: a novel approach for accurate taxonomic classification of microbiome sequences. Microbiome 6:140. https://doi.org/10.1186/s40168-018-0521-5

[35] Wang Q, Garrity GM, Tiedje JM, Cole JR (2007) Naive Bayesian classifier for rapid assignment of rRNA sequences into the new bacterial taxonomy. Appl Environ Microbiol. 73:5261-7. doi: 10.1128/AEM.00062-07.

[36] Deshpande V, Wang Q, Greenfield P, Charleston M, Porras-Alfaro A, Kuske CR, Cole JR, Midgley DJ, Tran-Dinh N (2016) Fungal identification using a Bayesian classifier and the Warcup training set of internal transcribed spacer sequences. Mycologia 108:1-5. doi: 10.3852/14-293.

[37] Oksanen FJ, Blanchet G, Friendly M, Kindt R, Legendre P et al (2020) Vegan: Community Ecology Package. R package version 2.5-7. https://CRAN.R-project.org/package=vegan
